# Rectal ozone insufflation modulates gut microbiota: improving lung function in mice with chronic obstructive pulmonary disease

**DOI:** 10.4103/mgr.MEDGASRES-D-25-00314

**Published:** 2026-09-12

**Authors:** Qinyao Zhao, Xiaofang Zhu, Pengcheng Wang, Shuangjiao Cao, John P. Williams, Jianxiong An

**Affiliations:** 1 Laboratory of Anesthesia and Critical Care Medicine in Colleges and Universities of Shandong Province, School of Anesthesiology, Shandong Second Medical University, Weifang, Shandong Province, China; 2 Laboratory of Ozone Medicine and Institute for Innovation Diagnosis & Treatment in Anesthesiology, Shandong Second Medical University, Weifang, Shandong Province, China; 3 Department of Anesthesiology and Perioperative Medicine, University of Pittsburgh School of Medicine, Pittsburgh, PA, USA; 4 Department of Anesthesiology for the Integrated Traditional Chinese and Western Medicine Hospital, Southern Medical University, Guangzhou, Guangdong Province, China

**Keywords:** acetic acid, Akkermansia, chronic obstructive pulmonary disease, fecal microbial transplantation, gut microbiota, gut–lung axis, iso-butyric acid, iso-pentanoic acid, lung function, ozone rectal insufflation, short-chain fatty acid

## Abstract

Ozone rectal insufflation possesses anti-inflammatory and antioxidant effects and is used for the treatment and prevention of diseases. This study aimed to investigate the effects of ozone rectal insufflation on chronic obstructive pulmonary disease and explore the potential underlying mechanisms from the perspective of gut microbiology. A mouse chronic obstructive pulmonary disease model was established by intratracheal spraying of lipopolysaccharide and elastase. Mice received ozone rectal insufflation intervention following modeling. We found that ozone rectal insufflation improved pulmonary function, alleviated emphysema and pulmonary fibrosis, and reduced inflammatory cytokine levels in the bronchoalveolar lavage fluid of chronic obstructive pulmonary disease mice. Additionally, ozone rectal insufflation modulated the pathogenic-to-beneficial gut bacteria ratio in chronic obstructive pulmonary disease mice. The treatment also led to an upregulation of short-chain fatty acids. The fecal microbiota transplantation results further confirmed that the gut microbiota of ozone rectal insufflation-treated mice was sufficient to attenuate chronic obstructive pulmonary disease symptoms. In summary, our findings indicate that ozone rectal insufflation enhances lung function and reduces inflammation in chronic obstructive pulmonary disease mice by modulating the gut microbiota and its associated metabolites.

## Graphical abstract

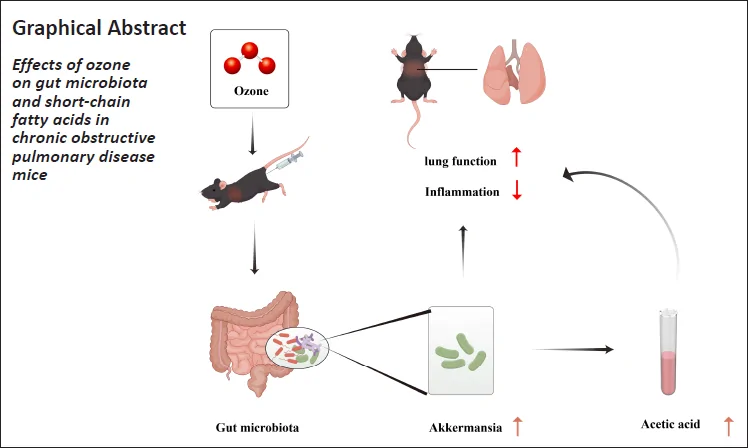

## Introduction

The incidence and mortality associated with chronic respiratory diseases continue to rise, primarily due to chronic obstructive pulmonary disease (COPD).^1^ COPD typically affects the function of multiple systems.^2^ Conventional pharmacotherapies frequently offer only modest efficacy and impose non-negligible adverse-event burdens, underscoring the urgent imperative to develop equally potent yet markedly safer therapeutic alternatives.

Ozone (O_3_) is a reactive gas with three oxygen atoms, typically derived from molecular oxygen.^3^,^4^ Medical ozone is available in several clinical forms, including ozonated oil, ozonated water, and ozone gas.^5^ Currently, oxygen–O_3_ therapy is a well-established medical practice with few documented adverse effects. It is used to treat a variety of conditions, such as cardiovascular, dermatological, and gastrointestinal disorders.^6^,^7^,^8^,^9^ There are relatively few studies on the mechanisms underlying systemic ozone application for disease. Generally, there are two primary pathways: one involves the direct effects on the local environment, including antibacterial and antiviral activities as well as the regulation of intestinal microorganisms. Another pathway involves the absorption of O_3_ reaction products into the bloodstream, where they exert anti-inflammatory and antioxidant effects.^10^,^11^ A study has shown that O_3_ inhalation leads to pulmonary inflammation.^12^ Therefore, inhalation has long been regarded as a contraindication to O_3_ therapy. While most studies have employed intraperitoneal injection of liquid O_3_, O_3_ rectal insufflation (O_3_-RI) has become increasingly common in clinical practice due to its non-invasive nature. This technique involves the administration of gaseous O_3_ into the rectum for therapeutic purposes, serving as an effective adjunctive therapy.^13^ A recent advancement in O_3_-RI is its application in the treatment of lung diseases. For example, in a case-control study, 14 patients with coronavirus disease 2019 were treated with O_3_-RI. The O_3_ protocol consisted of 8 sessions (1 session/day) of intra-rectal O_3_ (150 mL volume, 35 μg/mL concentration). The results indicated O_3_-RI improved the severity of a patient with coronavirus disease 2019 viral pneumonia.^14^ Another animal study found that O_3_-RI attenuated acute lung injury.^15^

There is a bi-directional interaction between the lungs and the intestines, known as the gut-lung axis.^16^ The intestinal microecosystem exists in a symbiotic relationship within the gut.^17^ Hypoxia, inflammatory response, oxidative stress, and dysregulation of the gut microbiota are considered prominent contributors to gut-lung interactions.^18^ Mice infected with *Heligmosomoides polygyrus* bakeri exhibited alterations in the structure and function of their intestinal microbiota, resulting in increased production of short-chain fatty acid (SCFA), which in turn led to a reduction in allergic airway inflammation.^19^ Additionally, most COPD patients exhibit gastrointestinal symptoms.^20^ Although no distinct gut-microbial signature has yet been linked to COPD onset, it is believed that gut microbiota may impact COPD through immune modulation via intestinal inflammation.

A previous study has demonstrated that O_3_-RI can alleviate cognitive dysfunction in mice via modulation of the gut microbiota.^10^ A previous clinical study has demonstrated the positive impact of O_3_ treatment on patients with gut dysbiosis,^21^ indicating that the modulation of gut microbiota could be a potential mechanism of O_3_-RI. However, the effects of O_3_-RI on COPD-induced pulmonary dysfunction remain unclear. Therefore, this study aimed (1) to explore the effects of O_3_-RI on COPD-related lung dysfunction and inflammation and (2) to investigate and verify whether O_3_-RI improves lung function and inflammation in COPD mice by modulating gut microbiota.

## Methods

### Animals and study design

Forty-eight 7-week-old male C57BL/6J mice, weighing between 22 and 25 g, were obtained from Jinan Pengyue Laboratory Animal Breeding Co., Ltd. (Jinan, Shandong, China, animal license No. SCXK(Lu)20210006). The mice were housed in the Experimental Animal Center of Shandong Second Medical University (animal license No. SYXK(Lu)20240026). The study was approved by the Ethics Committee for Laboratory Animal Experiments of Shandong Second Medical University (approval No. 2023SDL369, approval date November 16, 2023). All procedures complied with the Guide for the Care and Use of Laboratory Animals,^22^ ensuring strict adherence to rigorous laboratory protocols. Each of the six mice was individually housed in a plastic cage under controlled conditions, with the ambient temperature maintained at approximately 25°C. Sufficient food and water were provided throughout the study period.

This experiment was conducted in two parts (**Figure 1A** and **B**). Part 1 investigates the efficacy of O_3_ rectal infusion in a mouse COPD model. A total of 24 mice were allocated at random into four groups: Control (*n* = 6), COPD (*n* = 6), low-concentration O_3_-RI (O_3_-RI-L, *n* = 6), and high-concentration O_3_-RI (O_3_-RI-H, *n* = 6) groups. Part 2 aims to validate whether O_3_ rectal infusion improves the mouse COPD model via gut microbiota through fecal microbial transplantation (FMT). Another 24 mice were randomly divided into four groups: COPD (*n* = 6), low-concentration O_3_-RI + COPD (O_3_-RI-L+ COPD, *n* = 6), FMT + COPD (*n* = 6), and fecal microbiota transplantation-low concentration O_3_-RI (FMT + O_3_-RI-L + COPD, *n* = 6) groups.

**Figure 1:**
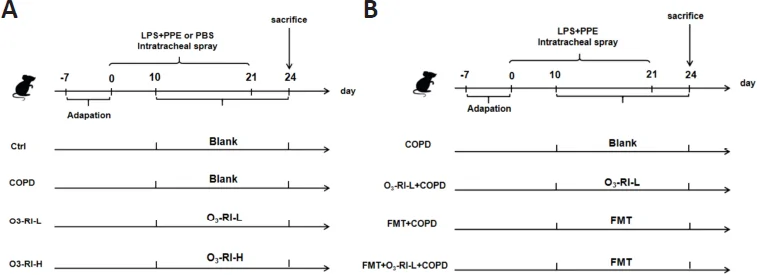
Experimental design. (A) Part 1. (B) Part 2. COPD: Chronic obstructive pulmonary disease; Ctrl: Control; ELT: porcine pancreatic elastase; FMT: fecal microbiota transplantation; LPS: lipopolysaccharide; O_3_-RI: ozone rectal insufflation; O_3_-RI-H: high-concentration O_3_-RI; O_3_-RI-L: low-concentration O_3_-RI.

### Chronic obstructive pulmonary disease model establishment

A mouse COPD model was established by intratracheal spraying 50 μL of a mixture containing 7 μg lipopolysaccharide (LPS; Sigma, St. Louis, MO, USA, L2880)and 1.2 U porcine pancreatic elastase (ELT; MCE, Monmouth Junction, NJ, USA, HY-P2974) once a week for 4 weeks.^23^ Animals in the control group received 50 μL of phosphate buffer saline using the same intratracheal spray method over the same duration. Intratracheal spraying was performed using a micro-nebulizer (Shanghai TOW Intelligent Technology Co., Ltd., Shanghai, China).^24^

### Ozone rectal insufflation treatment

O_3_ gas was produced by an O_3_ generator (Tri-Oxide Technology Co., Ltd., Jinan, China). Following the protocol established as previously reported, O_3_ concentrations of 10 μg/mL (low concentration) or 20 μg/mL (high concentration) were administered at a dose of 1.1 mg/kg per day.^25^ On the 10^th^ day, the antioxidant cannula was slowly placed in the rectum of mice, and O_3_ gas was injected. The average O_3_ injection volumes for the low- and high-concentration groups were approximately 2.64 and 1.32 mL, respectively. The O_3_-RI treatment was repeated daily for 14 days. In contrast, the control and COPD groups underwent the same cannula insertion procedure but did not receive O_3_-RI treatment.

### Lung function test

The lung function was evaluated using the eSpira™ Forced Manoeuvers System (EMMS, Bordon, Hampshire, UK).^26^ Following deep anesthesia with 1% sodium pentobarbital (45 mg/kg, intraperitoneal injection; Merck, Darmstadt, Germany), an incision was made in the skin of the mouse’s neck, and the trachea was exposed. An incision was then made in the upper portion of the cartilaginous ring of the trachea, allowing for the insertion of a tracheal catheter. After the pressure transducer, pneumatic transducer, and animal were properly connected, measure the mouse’s functional residual capacity (FRC), forced expiratory volume in 100 ms (FEV100), forced expiratory volume in 100 ms/forced vital capacity (FEV100/FVC), and dynamic compliance.^27^

### Enzyme-linked immunosorbent assay for bronchoalveolar lavage fluid extraction

Mice were anesthetized using 1% sodium pentobarbital. A catheter was inserted into the trachea, and the lungs were irrigated three times with 500 μL of pre-cooled phosphate buffer saline to obtain bronchoalveolar lavage fluid (BALF). The collected liquid was centrifuged for 5 minutes (4°C, 54 × *g*). The supernatant was aspirated, aliquoted, and stored at −80°C. Concentrations of tumor necrosis factor-alpha (TNF-α), interleukin (IL)-6 and IL-1β in BALF were measured using enzyme-linked immunosorbent assay kits (Elabscience Biotechnology Co., Ltd., Wuhan, China).^28^

### Histopathological assessment

After being anesthetized with 1% sodium pentobarbital, the left lung tissue was fixed in 4% paraformaldehyde for 48 hours before being embedded in paraffin. To evaluate lung inflammation and fibrosis, paraffin-embedded sections (4 μm thick) were stained with hematoxylin and eosin (HE) staining (Servicebio, Wuhan, China), Masson’s trichrome staining (Servicebio), and Victoria blue (VB) staining techniques (Servicebio). The severity of emphysema was quantified by measuring the mean linear intercept using HE-stained sections, with analysis performed via Image Pro Plus (Media Cybernetics, New York, NY, USA) from the HE stained sections. Additionally, Masson trichrome and VB staining sections were utilized for quantitative analysis. The area occupied by collagen or elastic fibers was quantified using Image-Pro Plus software, then divided by the total area examined (percentage of collagen fibers or percentage of elastic fibers).^29^,^30^,^31^

### 16S rRNA gene sequencing

Mouse fecal genomic DNA was extracted using the OMEGA Soil DNA Kit (M5635-02, Omega Bio-Tek, Norcross, GA, USA). The quantity and quality of the DNA were assessed using a NanoDrop NC2000 spectrophotometer (Thermo Fisher Scientific, Waltham, MA, USA) and agarose gel electrophoresis, respectively. For amplification of the V3-V4 region of the 16S rRNA gene, the forward primer 338F (5’-ACT CCT ACG GGG AGG CAG CA-3’) and reverse primer 806R (5’-GGA CTA CHV GGT WTC TAA T-3’) were used.32,33 The polymerase chain reaction products were purified using VazymeVAHTSTM DNA Clean Beads (Vazyme, Nanjing, China) and quantified using Quant-iT PicoGreen dsDNA detection kit (Invitrogen Inc., Carlsbad, CA, USA). The sequence data was mainly processed using the QIIME2 (https://qiime2.org/)^34^ and R software packages (v3.2.0). Briefly, raw sequence data were demultiplexed using the demux plugin following primer cutting with the cutadapt plugin.^35^ Sequences were then quality filtered, denoised, merged, and chimera removed using the DADA2 plugin.^36^ Non-singleton amplicon sequence variants were aligned with mafft^37^ and used to construct a phylogeny with fasttree2.^38^ α-Diversity metrics (Chao1,^39^ Observed species, Shannon^40^) and β-diversity metrics (weighted UniFrac,^41^ unweighted UniFrac,^42^ Jaccard distance, and Bray-Curtis dissimilarity) were estimated using the diversity plugin with samples that were rarefied by Precision Medicine & Translational Metabolomics Co., Ltd. (Shanghai, China). Linear discriminant analysis score > 2 serves as an empirical threshold that balances the mathematical significance of a 100-fold abundance difference on a logarithmic scale with the practical goal of identifying key biomarkers.

### Quantitative short-chain fatty acid measurement

Mouse fecal metabolite extraction was performed using a Biomek 4000 workstation (Beckman Coulter, Inc., Brea, CA, USA). Freshly prepared derivatization reagents were added to each well, and the plates were blocked for derivatization (MSC-100, Quan Sheng Instrument Co., Ltd., Hangzhou, China). The samples were then diluted with methanol solution in an ice bath and incubated at −20°C for 20 minutes. After centrifugation, 135 μL of supernatant was collected and transferred to a new 96-well plate. SCFAs were quantified using ultra-performance liquid chromatography-tandem mass spectrometry. Raw data files were processed using MassLynx (v4.1, Waters, Milford, MA, USA), including integration, calibration, and quantification of each metabolite peak. Subsequent statistical analyses, such as Principal Component Analysis, Orthogonal Partial Least Squares Discrimination Analysis, and univariate statistical tests, were performed using in-house developed iMAP software (v1.0, Metabo-Profile, Shanghai, China).

### Fecal microbial transplantation

Antibiotics (ampicillin, vancomycin, metronidazole, and neomycin) (all provided by MeilunBio, Dalian, China) were administered to the FMT groups through their drinking water for 5 consecutive days to deplete the gut microbiome.^23^ Subsequently, 200 mg of fecal matter was collected from mice in the COPD and O_3_-RI-L groups, respectively, and resuspended in 5 mL of phosphate buffer saline. The homogenate was filtered through a nylon filter (Bkmamlab, Changde, China), and the resulting suspension was centrifuged for 2 minutes (4°C, 2200 r/min). Mice in the FMT group were then gavaged with 200 μL of the supernatant twice weekly for 2 weeks.^29^

### Statistical analysis

The data were first tested for normality, with normally distributed numerical variables shown as mean and standard deviation and skewed numerical variables shown as median and interquartile range. Tukey’s multiple comparison test was then applied to further pairwise comparisons. Statistical analyses and data visualization were performed using GraphPad Prism 9.5.0 (Windows, GraphPad Software, Boston, MA, USA, www.graphpad.com). A *P*-value < 0.05 was considered statistically significant.

## Results

### O_3_-RI improves the lung function of COPD mice

To evaluate the effects of O_3_-RI on pulmonary dysfunction in COPD mice, lung function tests in Part 1 were performed. The results demonstrated that mice in the COPD group exhibited an increase in FRC and dynamic compliance, along with decreased FEV100/FVC and lower FEV100 (**Figure 2A−D**). Mice in the O_3_-RI group showed significant improvements in the above four lung function parameters compared with the COPD group (all *P* < 0.01).

**Figure 2:**
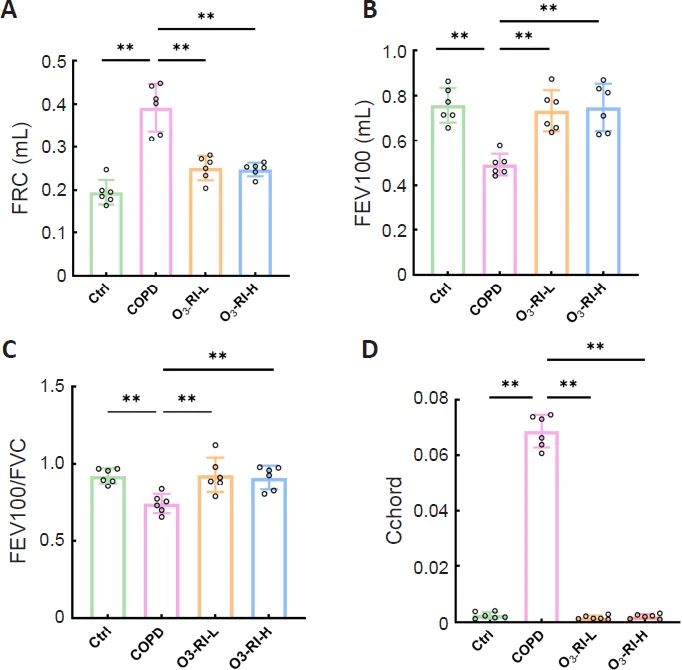
O_3_-RI improves lung function of COPD mice. (A) FRC. (B) FEV100. (C) FEV100/FVC. (D) Cchord. **P < 0.01 (one-way analysis of variance followed by the Tukey’s multiple comparison test). Cchord: Dynamic compliance; Ctrl: Control; COPD: chronic obstructive pulmonary disease; FEV100/FVC: functional residual capacity/forced vital capacity; FRC: functional residual capacity; O_3_-RI: ozone rectal insufflation; O_3_-RI-H: high-concentration O_3_-RI; O_3_-RI-L: low-concentration O_3_-RI.

### Ozone rectal insufflation reduces lung inflammation in chronic obstructive pulmonary disease mice

To evaluate the effect of O_3_-RI on pulmonary inflammation in COPD mice, inflammatory cytokine testing was conducted in part 1. The concentrations of the inflammatory cytokines in the BALF were analyzed by enzyme-linked immunosorbent assay (**Figure 3A−C**). Results indicated that the concentrations of TNF-α, IL-6 and IL-1β were significantly higher in the COPD group compared to the control group (*P* < 0.05 or *P* < 0.01). After O_3_-RI treatment, a significant decrease in the concentrations of these cytokines was observed (all *P* < 0.05 or *P* < 0.01).

**Figure 3:**
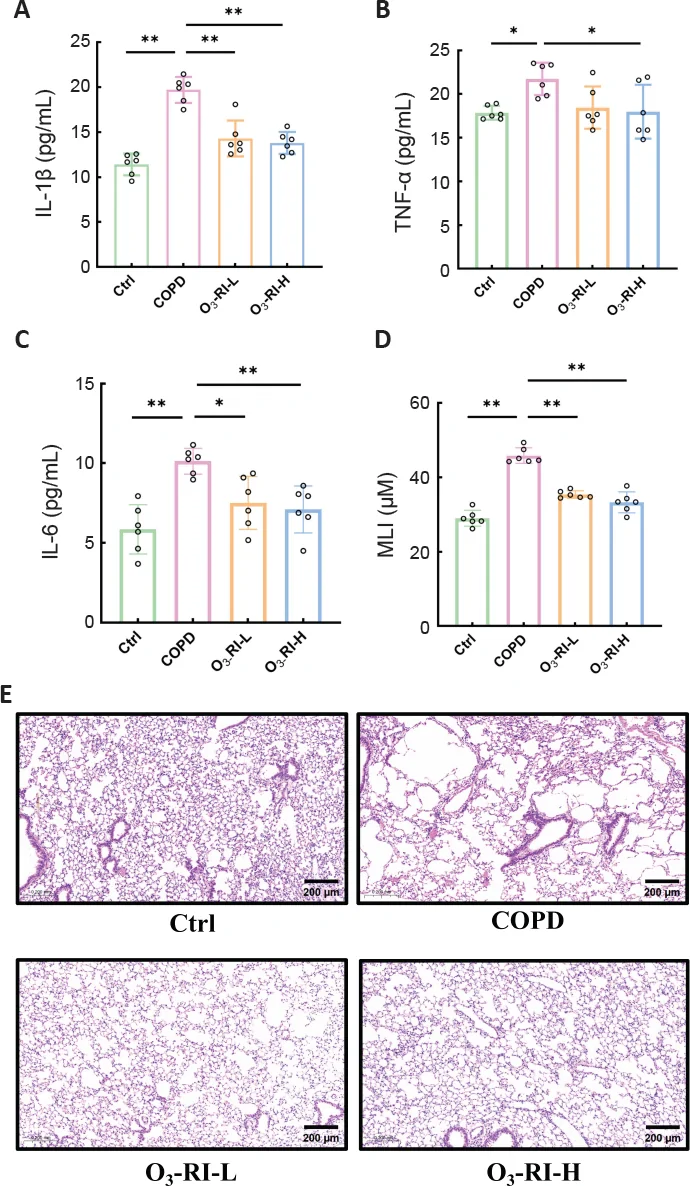
O_3_-RI improves emphysema and lung inflammation in COPD mice. (A–C) Levels of inflammatory cytokines in BALF measured by ELISA. (D) The severity of emphysema using the MLI method. (E) Representative HE staining of lung tissues (100× original magnification, scale bar: 200 μm). Compared with COPD, O_3_-RI showed marked improvement with no significant alveolar collapse observed, though occasional alveolar wall ruptures and fusions were noted. This suggests that O_3_-RI may have a certain ameliorative effect on alveolar damage caused by COPD. Scale bars: 200 μm. *P < 0.05, **P < 0.01 (one-way analysis of variance followed by the Tukey’s multiple comparison test). BALF: bronchoalveolar lavage fluid; COPD: chronic obstructive pulmonary disease; Ctrl: Control; ELISA: enzyme-linked immunosorbent assay; HE: hematoxylin and eosin; IL: interleukin; MLI: mean linear intercept; O_3_-RI: ozone rectal insufflation; O_3_-RI-H: high-concentration O_3_-RI; O_3_-RI-L: low-concentration O3-RI; TNF: tumor necrosis factor.

### Ozone rectal insufflation reduces lung tissue damage in chronic obstructive pulmonary disease mice

To evaluate the effects of O_3_-RI on the pathological morphology of the lungs in COPD mice, pathological section experiments were conducted in part 1. HE staining, Masson trichrome staining, and VB staining were used to assess the histopathological changes in lung tissue. The HE staining results showed that the COPD group exhibited an enlarged alveolar lumen, with an increased mean linear intercept. In contrast, the alveolar morphology was relatively intact in the O_3_-RI groups compared to the COPD group (**Figure 3D** and **E**). Masson’s trichrome and VB staining further revealed a substantially increased collagen fiber content in the airways and a reduction in elastic fibers within the alveolar walls in the COPD group. These pathological changes were notably alleviated in the O_3_-RI-L and O_3_-RI-H groups relative to the COPD group (**Figure 4A−D**).

**Figure 4:**
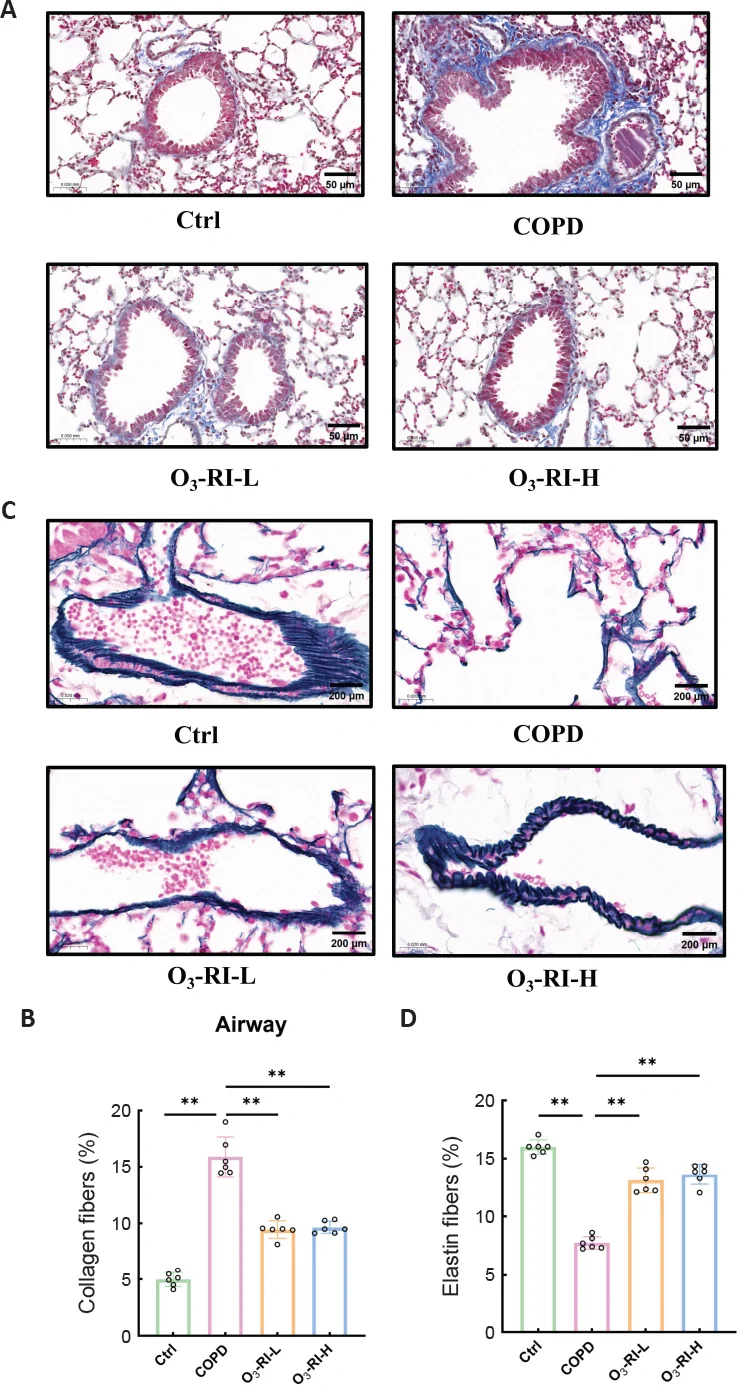
O_3_-RI ameliorates lung fibrosis in COPD mice. (A) Representative Masson trichrome of staining lung tissues. Compared with COPD, O_3_-RI significantly reduced the collagen deposition (blue-stained areas) and smooth muscle hyperplasia (red-stained areas), which suggests that O_3_-RI may alleviate collagen deposition and smooth muscle hyperplasia. (B) Quantitative analysis of lung fibrosis by Masson trichrome staining. (C) Representative VB staining of lung tissues from Ctrl, COPD, O_3_-RI-L, and O_3_-RI-H groups. Compared with COPD, the elastic fibers in the alveolar walls were elevated after O_3_-RI treatment, suggesting that O_3_-RI may help restore the reduced elastic fibers in the alveolar walls in COPD. Scale bars: 50 μm (A), 200 μm (C). (D) Quantitative analysis of lung fibrosis by VB staining. **P < 0.01 (one-way analysis of variance followed by Tukey’s multiple comparison test). COPD: chronic obstructive pulmonary disease; Ctrl: Control; O_3_-RI: ozone rectal insufflation; O_3_-RI-H: high-concentration O_3_-RI; O_3_-RI-L: low-concentration O_3_-RI; VB: Victoria blue.

### Ozone rectal insufflation affects gut microbiota diversity in chronic obstructive pulmonary disease mice

#### The α- and β-diversity analysis

To assess the diversity of the mouse gut microbiota, we conducted the α- and β-diversity analysis. The results showed a decrease in both bacterial abundance and diversity in the COPD group, as evidenced by significant differences in the Chao1 and Observed-species indices compared to the control group. Notably, O_3_-RI treatment led to an increase in microbial richness and diversity (**Figure 5A**). To evaluate β-diversity, principal coordinate analysis was conducted using the Jaccard distance to assess differences in microbial community composition across groups (**Figure 5B**). The two principal component scores accounted for 7.2% and 11.9% of the total variation in β-diversity, respectively. Principal coordinate analysis revealed distinct clustering patterns in gut microbiota composition, differentiating the COPD group from the control group. O_3_-RI significantly changed the composition of the intestinal microbiota.

**Figure 5:**
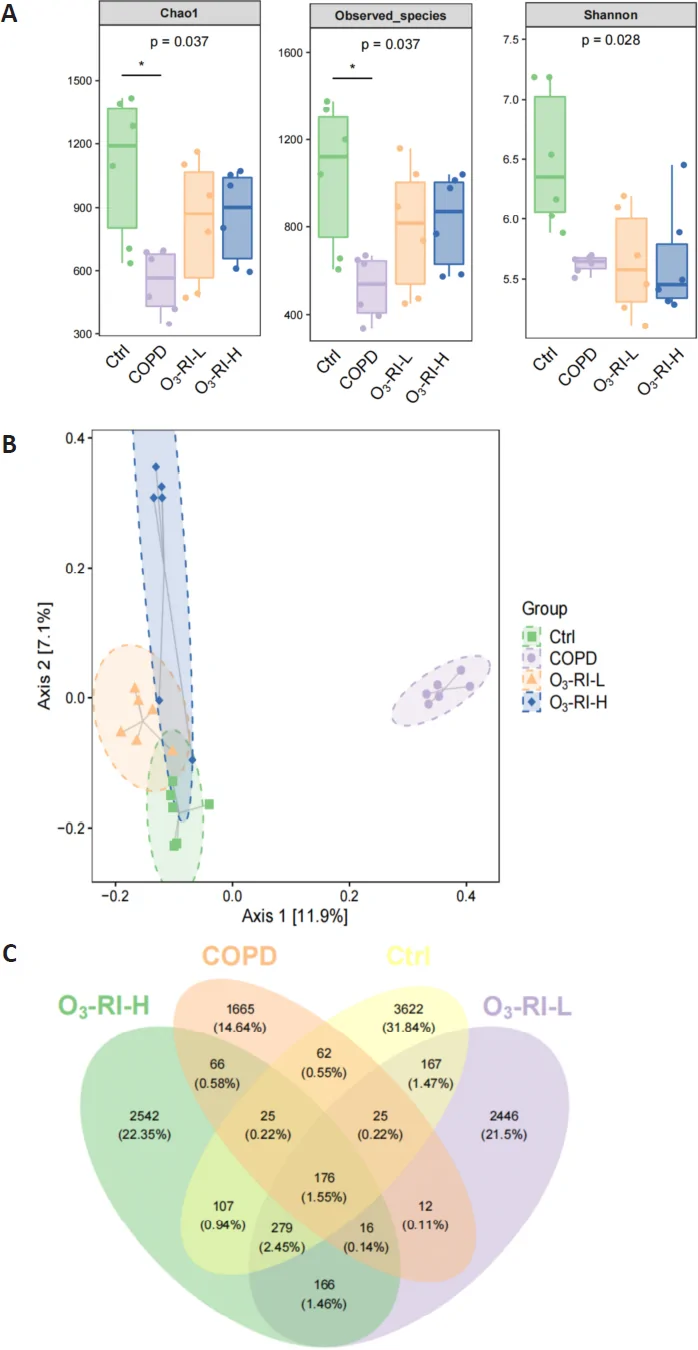
O_3_-RI affects the gut microbiota diversity in COPD mice. (A) Chao1, Observed-species and Shannon indices. (B) Principal coordinates analysis. (C) Venn diagram of similarity and overlap of operational taxonomic unit numbers in each group. COPD: chronic obstructive pulmonary disease; Ctrl: Control; O_3_-RI: ozone rectal insufflation; O_3_-RI-H: high-concentration O_3_-RI; O_3_-RI-L: low-concentration O_3_-RI.

#### The operational taxonomic units number analysis

To further investigate the specific characteristics of changes in microbial composition, a Venn diagram was employed to visualize the shared and unique operational taxonomic units (OTUs) among the groups. Although most OTUs were distributed across all groups, a unique OTU distribution pattern was observed between the COPD group and the O_3_-RI groups (administered at two different concentrations). The analysis revealed 1749 OTUs unique to the COPD group that were absent in the control group. In contrast, the O_3_-RI groups exhibited 2542 and 2446 unique OTUs for the low- and high-concentration treatments, respectively (**Figure 5C**).

#### The gut microbiota composition analysis

To assess the composition of the gut microbiota, we analyzed the makeup of the microbial community across two taxonomic ranks: phylum and genus. At the phylum level, the COPD group exhibited a significant decrease in the abundance of the Bacteroidetes and an increase in Firmicutes, whereas this trend was reversed by O_3_-RI-L, highlighting the regulatory effects of O_3_-RI on microbial community structure (**Figure 6A** and **B**). At the genus level, we identified the 20 most abundant genera to provide a more detailed characterization of the community structure (**Figure 6C**).

**Figure 6:**
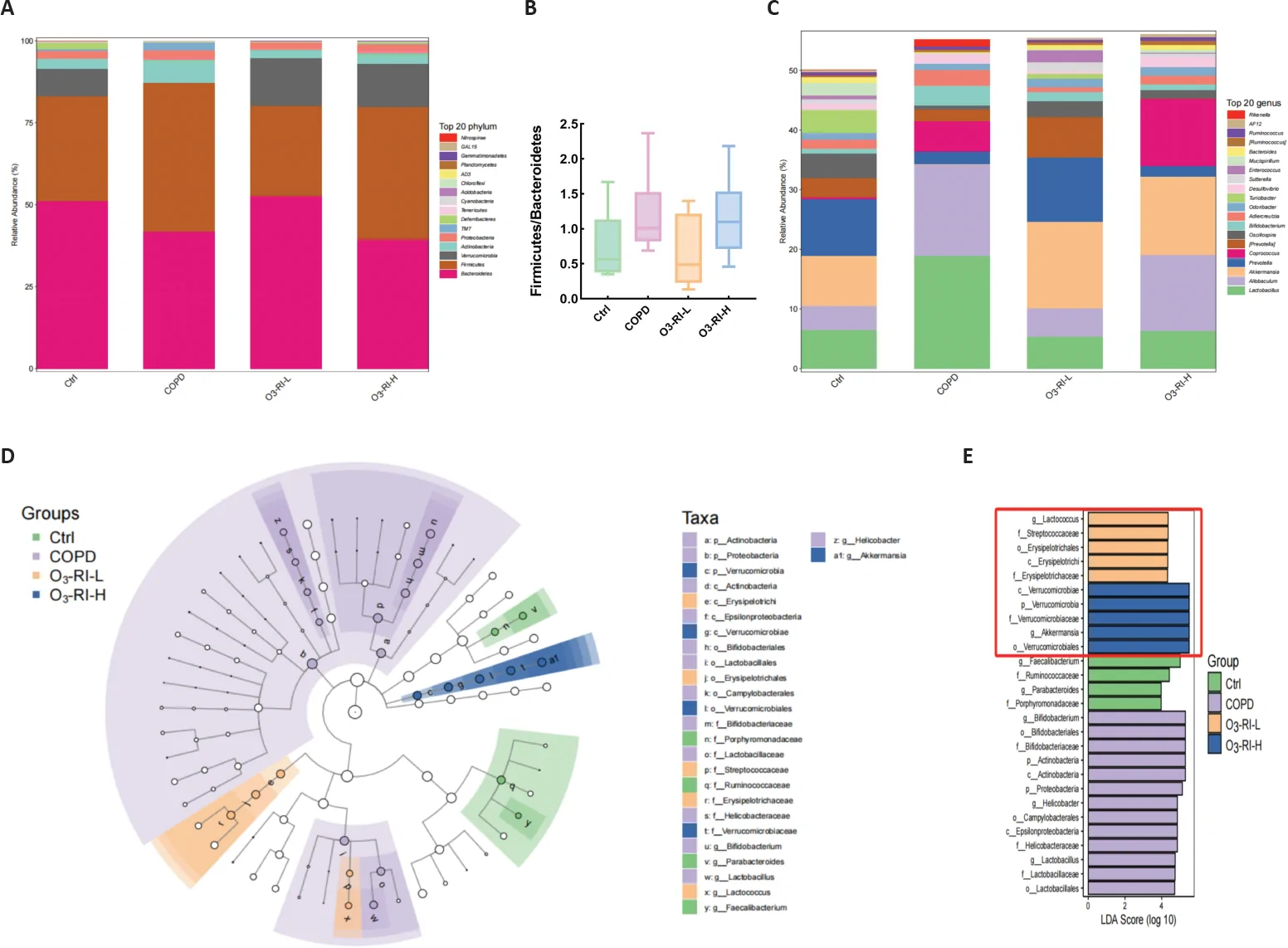
O_3_-RI affects the gut microbiota composition in COPD mice. (A) Gut microbiota makeup at the phylum taxonomic level. (B) The Firmicutes/Bacteroidetes ratio. (C) Gut microbiota makeup at the genus level. (D) Cladogram for taxonomy. (E) LDA scores of taxa (score > 2). COPD: Chronic obstructive pulmonary disease; Ctrl: Control; O_3_-RI: ozone rectal insufflation; LDA: linear discriminant analysis; O_3_-RI-H: high-concentration O_3_-RI; O_3_-RI-L: low-concentration O_3_-RI.

#### The linear discriminant analysis

To identify species exhibiting significant differences in abundance between groups, we performed linear discriminant analysis (**Figure 6D** and **E**). The LEfSe plots visualized these differences, identifying several biomarkers associated with the COPD group, including g_Bifidobacterium, o_Bifidobacteriales, f_Bifidobacteriaceae, p_Actinobacteria, c_Actinobacteria, p_Proteobacteria, g_Helicobacter, o_Campylobacterales, c_Epsilonproteobacteria, f_Helicobacteraceae, g_Lactobacillus, f_Lactobacillaceae, and o_Lactobacillales. In contrast, O_3_-RI treatment altered microbial biomarkers, such as g_Lactococcus, f_Streptococcaceae, o_Erysipelotrichales, c_Erysipelotrichi, f_Erysipelotrichaceae, c_Verrucomicrobiae, p_Verrucomicrobia, f_Verrucomicrobiaceae, g_Akkermansia, and o_Verrucomicrobiales were altered (linear discriminant analysis score > 2).

### Ozone rectal insufflation affects intestinal short-chain fatty acid concentration in chronic obstructive pulmonary disease mice

To assess changes in SCFAs, we conducted a metabolomics analysis in part 1. Metabolomics analysis showed that SCFAs were the predominant metabolites (**Figure 7A**). O_3_-RI significantly increased the isocaproic acid, butyric acid, acetic acid, and 2-methylpentanoic acid levels, as demonstrated by the SCFA marker analysis (**Figure 7B**). Pearson correlation analysis between gut microbiota and SCFA levels showed distinct relationships across the experimental groups. In the control group, Faecalibacterium was positively correlated with propionic acid. In the COPD group, Bifidobacteriaceae exhibited a negative correlation with the isoacetic acid. In the O_3_-RI-treated group, Akkermansia showed positive correlations with acetic acid, iso-butyric acid, and iso-pentanoic acid (**Figure 7C**).

**Figure 7:**
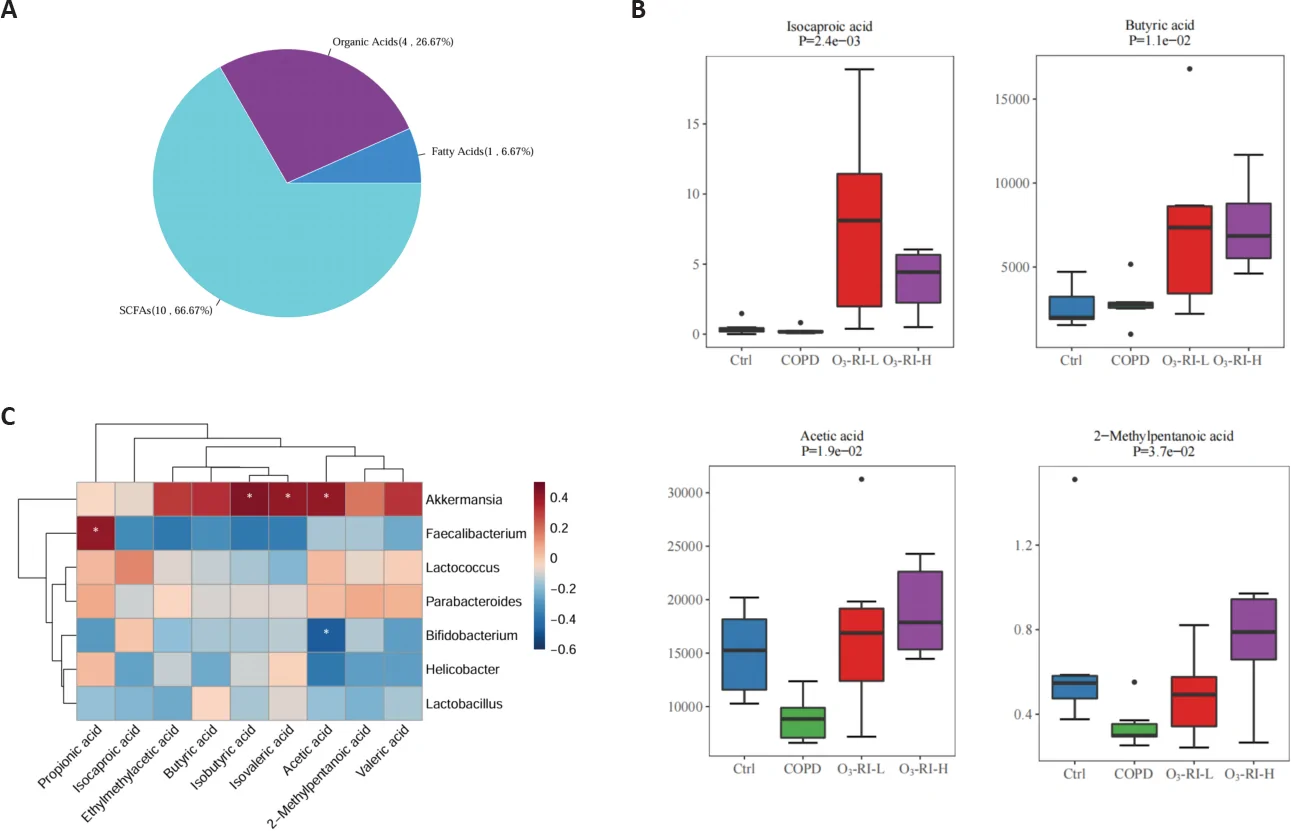
O_3_-RI affects the gut metabolite composition in COPD mice. (A) Gut metabolite composition. (B) SCFA analysis in the feces. (C) Pearson correlation analysis. *P < 0.05. COPD: Chronic obstructive pulmonary disease; Ctrl: Control; O_3_-RI: ozone rectal insufflation; O_3_-RI-H: high-concentration O_3_-RI; O_3_-RI-L: low-concentration O_3_-RI; SCFA: short-chain fatty acid.

### Fecal microbial transplantation improves lung function in chronic obstructive pulmonary disease mice

To evaluate the effects of fecal transplants on pulmonary dysfunction in COPD mice, lung function tests in part 2 were performed. FMT was administered to mice in the recipient group twice a week for 2 weeks, followed by an assessment of lung function across all groups. The results revealed that compared with the O_3_-RI-L + COPD group, mice in the FMT + COPD group exhibited elevated FRC and static compliance, along with decreased values of FEV100/FVC and FEV100 (*P* < 0.05 or *P* < 0.01). In contrast, compared with the COPD and FMT + COPD groups, the FMT + O_3_-RI-L + COPD group showed an improvement in these lung function indices (*P* < 0.05 or *P* < 0.01), indicating that fecal transplantation had a positive effect on lung function. Further analysis revealed that compared with the COPD group, the O_3_-RI-L + COPD group demonstrated greater improvements in FRC, static compliance, FEV100, and FEV100/FVC, outperforming the FMT + O_3_-RI-L + COPD group (*P* < 0.05 or *P* < 0.01) (**Figure 8A–D**).

**Figure 8:**
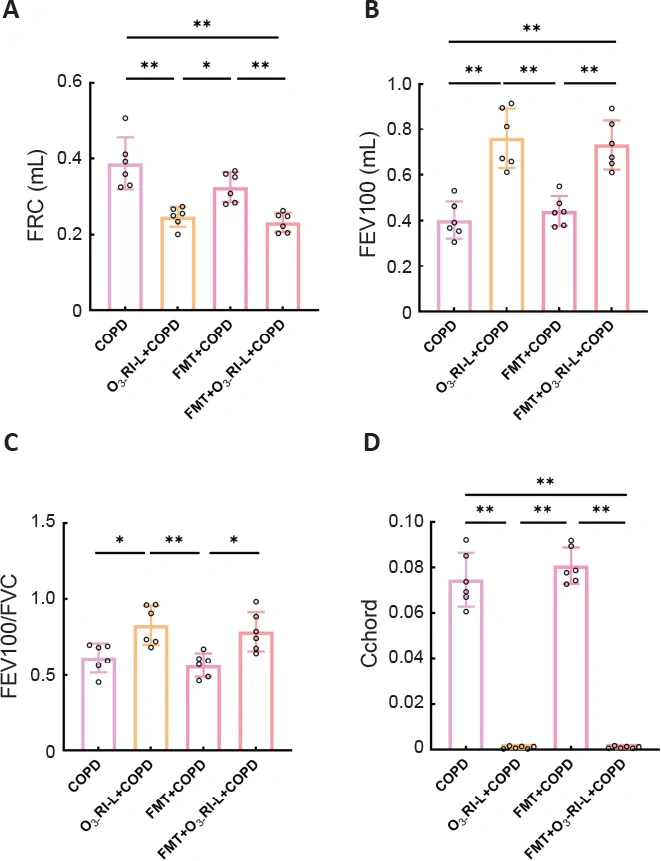
FMT improves lung function in COPD mice. (A) FRC. (B) FEV100. (C) FEV100/FVC. (D) Cchord. *P < 0.05, **P < 0.01 (one-way analysis of variance followed by Tukey’s multiple comparison test). Cchord: Dynamic compliance; COPD: chronic obstructive pulmonary disease; FEV100/FVC: functional residual capacity/forced vital capacity; FRC: functional residual capacity; FMT: fecal microbiota transplantation; O_3_-RI: ozone rectal insufflation; O_3_-RI-L: low-concentration O_3_-RI.

### Fecal microbial transplantation reduces lung inflammation in chronic obstructive pulmonary disease mice

To evaluate the effect of FMT on pulmonary inflammation in COPD mice, inflammatory cytokine testing was conducted in part 2. Compared with the O_3_-RI-L + COPD group, IL-1β, IL-6, and TNF-α were significantly increased in the BALF of mice in the FMT + COPD group (*P* < 0.05 or *P* < 0.01). In contrast, compared with the COPD and FMT + COPD groups, the FMT + O_3_-RI + COPD group exhibited significantly lower levels of these inflammatory cytokines (*P* < 0.05 or *P* < 0.01). Further analysis revealed that the O_3_-RI-L + COPD group was more effective than the FMT + O_3_-RI-L + COPD group in reducing inflammatory cytokines, with statistical significance (*P* < 0.05 or *P* < 0.01; **Figure 9A–C**).

**Figure 9:**
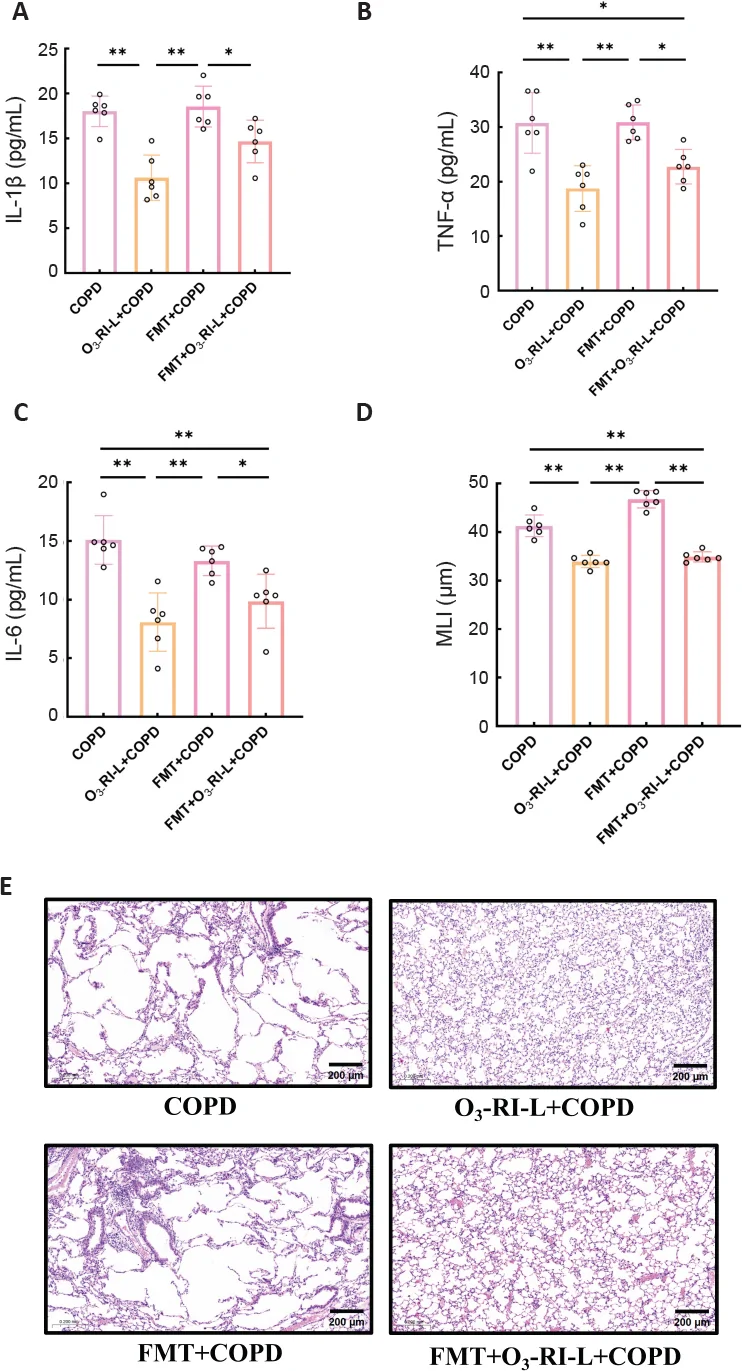
FMT improves emphysema and lung inflammation in COPD mice. (A-C) Levels of inflammatory cytokines in BALF measured by ELISA. (D) The severity of emphysema was quantified using the MLI method. (E) Representative HE staining of lung tissues (100× original magnification, scale bar: 200 μm). Compared with COPD, O_3_-RI-L and FMT-L treatment showed marked improvement in conditions such as variable alveolar sizes, ruptured and fused alveolar walls, alveolar collapse, enlarged alveolar spaces, and inflammatory cell infiltration. *P < 0.05, **P < 0.01 (one-way analysis of variance followed by the Tukey’s multiple comparison test). BALF: Bronchoalveolar lavage fluid; COPD: chronic obstructive pulmonary disease; ELISA: enzyme-linked immunosorbent assay; FMT: fecal microbiota transplantation; HE: hematoxylin and eosin; IL: interleukin; MLI: mean linear intercept; O_3_-RI: ozone rectal insufflation; O_3_-RI-L: low-concentration O_3_-RI; TNF: tumor necrosis factor.

### Fecal microbial transplantation reduces lung tissue damage in chronic obstructive pulmonary disease mice

To evaluate the effects of FMT on the pathological morphology of the lungs in COPD mice, pathological section experiments were conducted in part 2. Two weeks after fecal transplantation, we used HE staining, Masson trichrome staining, and VB staining to assess the pathological changes in the lung tissue of mice. The results revealed significant alveolar lumen enlargement in the FMT + COPD group, which led to an increase in mean linear intercept, reflecting a higher degree of structural damage to the lung tissues (**Figure 9D** and **E**). In contrast, the alveolar structures of mice in the FMT + O_3_-RI-L + COPD group were relatively well preserved, showing less pathological damage than that in the FMT + COPD group. Furthermore, Masson’s trichrome and VB staining showed that in the FMT + O_3_-RI-L + COPD group, collagen fiber content in the airways was significantly increased, while elastic fibers in the alveolar walls were decreased compared to the O_3_-RI-L + COPD group. Notably, all these indices were improved in the FMT + O_3_-RI-L + COPD group relative to the FMT + COPD group (**Figure 10A–D**).

**Figure 10:**
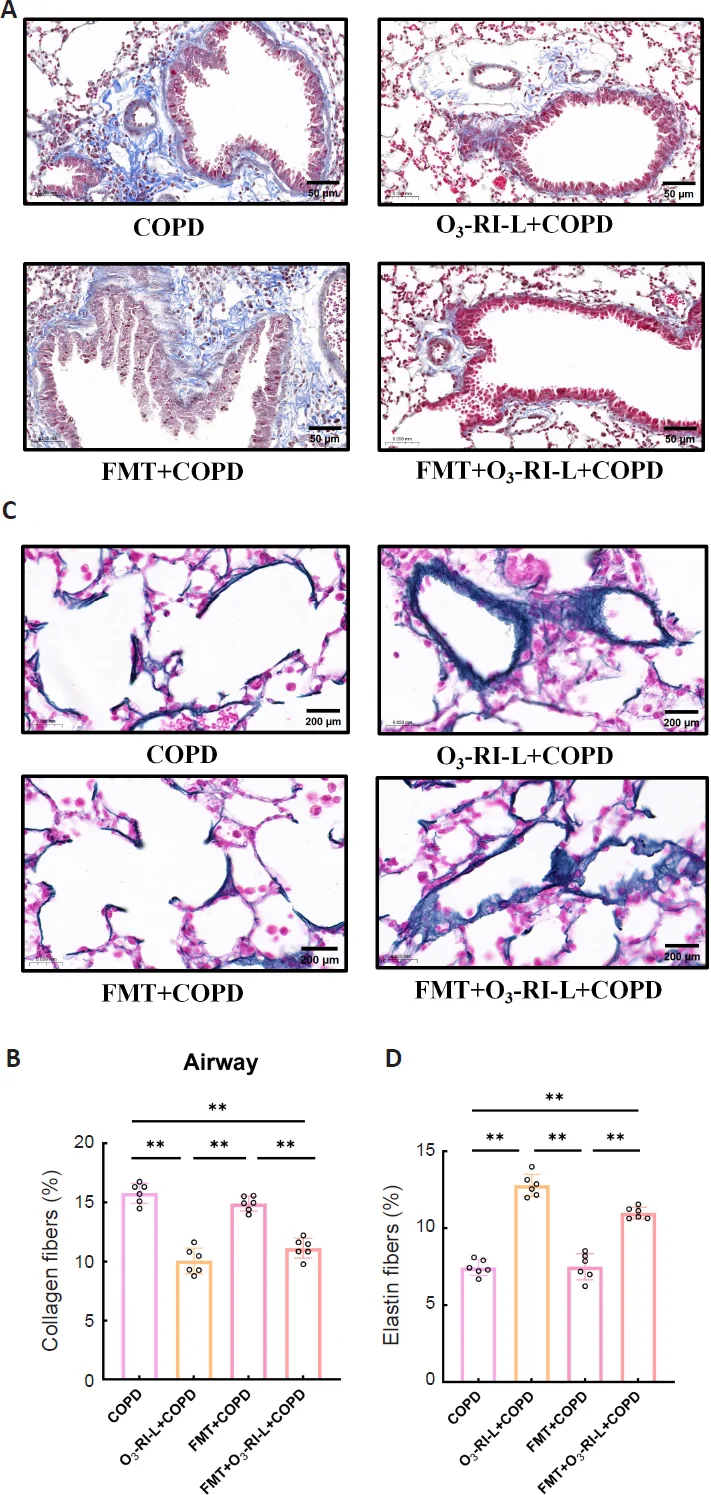
FMT ameliorates lung fibrosis in COPD mice. (A) Representative Masson trichrome staining of lung tissues (400× original magnification, scale bar: 50 μm). Compared with COPD, O_3_-RI-L and FMT-L significantly reduced the collagen deposition (blue-stained areas) and smooth muscle hyperplasia (red-stained areas), suggesting that they may alleviate collagen deposition and smooth muscle hyperplasia. (B) Quantitative image analysis of lung fibrosis by Masson trichrome staining. (C) Representative VB staining of lung tissues (400× original magnification, scale bar: 50 μm). Compared with COPD, O_3_-RI-L and FMT-L elevated the elastic fibers in the alveolar walls. *P < 0.05, **P < 0.01 (one-way analysis of variance followed by Tukey’s multiple comparison test). COPD: Chronic obstructive pulmonary disease; FMT: fecal microbiota transplantation; O_3_-RI: ozone rectal insufflation; O_3_-RI-L: low-concentration O_3_-RI; VB: Victoria blue.

## Discussion

Our study shows that LPS and ELT-induced COPD in mice results in significant pulmonary dysfunction and inflammation. However, these effects were mitigated upon administration of O_3_-RI. To validate the efficacy of O_3_-RI, we conducted several experimental analyses, including lung function tests, pathological staining, and inflammatory factor assays, across all groups. The results indicated that O_3_-RI improved both the pathological changes and inflammatory responses in the lungs of COPD mice. Two concentrations of O_3_ were used for therapeutic intervention; however, the therapeutic effects of the two concentrations did not differ significantly across most of the study outcomes. This suggests that the therapeutic efficacy of O_3_-RI may not be directly proportional to its concentration. Notably, in the gut microbiota analysis, we observed a reduction in the abundance and diversity of gut microbiota in COPD mice, which was reversed in the O_3_-RI-treated mice. Additionally, metabolite analysis revealed decreased SCFAs in COPD mice, whereas SCFA levels were upregulated following O_3_-RI treatment. These findings suggest that this study offers insights into the potential mechanisms through which O_3_-RI may alleviate respiratory diseases such as COPD.

Studies have emphasized the link between gut microbiota and its associated metabolites with COPD. As part of our research objectives, we analyzed the differences in gut microbiota composition across groups. Our results showed that the Chao1 and observed-species indices, which reflect gut microbiota (α-diversity), were significantly reduced in COPD mice compared to controls. This finding is consistent with a previous study.^43^ According to existing literature,^44^ reduced gut microbial diversity is associated with a higher prevalence of allergic diseases in children. Compared to the COPD group, the O_3_-RI group exhibited a significantly higher level of microbial diversity. β-Diversity analysis further supported the emergence of unique microbial communities in the COPD group, with the gut microbiota of O_3_-RI-treated mice trending toward a composition similar to that of the control group. This observation suggests that O_3_-RI has a beneficial effect in attenuating COPD-induced dysbiosis of the intestinal microbiota, thereby reinforcing the therapeutic potential of O_3_-RI in improving COPD conditions.

At the phylum level, our analyses revealed an elevated abundance of *Firmicutes* and a decreased abundance of *Bacteroidetes* in the gut microbiota of COPD mice. Notably, the major phyla in the gut microbiota are *Firmicutes* and *Bacteroidetes*.^45^ The imbalance between these two phyla has been well-documented in various respiratory disorders and clinical conditions.^46^,^47^ For example, in lung disease, the gut microbiota is characterized by an increased abundance of Firmicutes along with a decreased abundance of Bacteroidetes. Notably, our study demonstrated that O_3_-RI significantly reversed the Firmicutes/Bacteroidetes ratio, restoring it closer to the levels observed in the control group. Moreover, this recovery was more significant in the O_3_-RI-H group compared to the O_3_-RI-L group.

At the genus level, our analysis revealed a significantly lower abundance of *Prevotella* in the COPD group. As a member of the phylum *Bacteroidetes, Prevotella* is associated with the synthesis of SCFAs, which play crucial roles in anti-inflammatory and antimicrobial activities.^48^ Notably, the genus *Prevotella intestinalis* has been identified as a key participant in host-microbiome interactions, particularly concerning nutrition and metabolism.^49^ The interaction of *Prevotella* with mucosal immunity may explain its involvement in autoimmunity and its role in enhancing protection against pathogenic bacteria.^50^,^51^ Consistent with previous studies, the reduction in *Bacteroidetes*, especially *Prevotella*, is a characteristic feature of COPD-associated gut dysbiosis.^52^ Conversely, the genus *Lactobacillus* showed a significantly increased abundance in the COPD group.^52^ Lactobacillus, a common bacterial genus in the human gastrointestinal and reproductive tracts,^53^,^54^,^55^ has been reported to exhibit increased prevalence in patients with gastric cancer.^56^ This finding is notable, as gastroesophageal reflux, a known risk factor for COPD comorbidities, has been implicated in such microbial shifts.^57^,^58^,^59^,^60^ Furthermore, recent studies suggest that *Lactobacilli* modulate the immune response to influenza A virus in mice.^61^ The increased abundance of *Lactobacillus* in lung tissue could indicate its role as a target or immunomodulator contributing to the inflammatory response.^52^ In addition, O_3_-RI treatment significantly upregulated the abundance of other genera, including *Akkermansia muciniphila*. Since the discovery of *Akkermansia muciniphila* 20 years ago, numerous studies have linked its deficiency to obesity, diabetes, hepatic steatosis, inflammation, and tumor immunotherapy response.^62^ *Akkermansia muciniphila* plays a critical role in maintaining intestinal barrier integrity, modulating immune responses, and limiting inflammation, processes that underlie many diseases.^62^ Importantly, *Akkermansia muciniphila* has been shown to alleviate acute lung injury in mice by regulating gut microbiota and SCFA levels.^63^ These findings highlight the inextricable influence of gut microbiota, particularly genera such as *Prevotella*, *Lactobacillus*, and *Akkermansia*, on lung diseases, including COPD. These microbial shifts further underscore the potential of O_3_-RI as a therapeutic intervention.

Changes in gut SCFAs, induced by changes in the gut microbiota composition, have been shown to play a significant role in COPD.^64^ We examined the SCFA levels in the feces of mice and found that O_3_-RI significantly upregulated isocaproic acid, butyric acid, acetic acid, and 2-methylpentanoic acid in COPD mice. In addition, we explored the relationship between the intestinal microbiota and SCFAs. Notably, our findings revealed that within the different microbiota genera, the marker species *Faecalibacterium* in the control group was positively correlated with propionic acid, while *Akkermansia* in the O_3_-RI group was positively correlated with beneficial acids, including acetic acid, isobutyric acid, and isovaleric acid. In contrast, the marker species *Bifidobacteriaceae* in the COPD group showed a negative correlation with acetic acid. We observed changes in the structure and composition of the gut microbiota in the COPD group, with specific genera associated with a decrease in SCFAs. Importantly, O_3_-RI reversed these changes. Additionally, FMT from O_3_-RI-treated mice into COPD mice resulted in improved lung function and a reduction in lung inflammation. These results underscore the potential of O_3_-RI in alleviating COPD by modulating specific gut microbiota genera and SCFA production in COPD mice.

In the previous section, we found that O_3_-RI alleviated inflammation and dysfunction in LPS- and ELT-induced COPD mice by modulating the gut microbiota. To further verify this, we performed fecal microbiota transplantation to alter the microbiota of COPD mice. The results showed that the gut microbiota from O_3_-RI-treated mice ameliorated lung inflammation and dysfunction in COPD mice.

However, our exploratory study also has certain limitations. The inflammatory pathways through which O_3_-RI exerts its effects in the gut remain unexplored due to the complexity and diversity of O_3_-RI responses in the gut. The LPS combined with the ELT model used is an acute chemically induced model and does not fully replicate the chronic and progressive nature of human COPD. Female mice were not employed for validation. Furthermore, the experiment employed the gavage transplantation method and did not screen for acid-resistant bacteria. Future studies are needed to further investigate the positive effects of specific microbial communities and metabolites on COPD.

In conclusion, our findings indicate that O_3_-RI enhances lung function and reduces inflammation in COPD mice by modulating the gut microbiota and its associated metabolites. This research provides novel insights into the field of O_3_-RI and offers potential directions for its clinical application in the management of COPD.

## Data Availability

*All relevant data are within the paper.*
